# Antitumor in situ vaccination effect of TNFα and IL-12 plasmid DNA electrotransfer in a murine melanoma model

**DOI:** 10.1007/s00262-018-2133-0

**Published:** 2018-02-21

**Authors:** Urska Kamensek, Maja Cemazar, Ursa Lampreht Tratar, Katja Ursic, Gregor Sersa

**Affiliations:** 1 Department of Experimental Oncology, Institute of Oncology Ljubljana, Zaloska 2, 1000 Ljubljana, Slovenia; 2 Faculty of Health Sciences, University of Primorska, Polje 42, Izola, Slovenia; 3 Faculty of Health Sciences, University of Ljubljana, Zdravstvena pot 5, 1000 Ljubljana, Slovenia

**Keywords:** In situ vaccination effect, Gene electroporation, Interleukin 12, Tumor necrosis factor alpha, Murine melanoma, Vitiligo

## Abstract

Gene electrotransfer (GET) is one of the most efficient non-viral gene therapy approaches for the localized transfer of multiple genes into tumors in vivo; therefore, it is especially promising for delivering different cytokines that are toxic if administered systemically. In this study, we used concomitant intratumoral GET of two cytokines: tumor necrosis factor alpha (TNFα), a potent cytotoxic cytokine to induce in situ vaccination, and interleukin 12 (IL-12), an immunostimulatory cytokine to boost the primed local immune response into a systemic one. After performing GET in murine melanoma tumors, both TNFα and IL-12 mRNA levels were significantly increased, which resulted in a pronounced delay in tumor growth of 27 days and a prolonged survival time of mice. An antitumor immune response was confirmed by extensive infiltration of immune cells in the tumor site, and expansion of the effector immune cells in the sentinel lymph nodes. Furthermore, the effect of in situ vaccination was indicated by the presence of vitiligo localized to the treatment area and resistance of the mice to secondary challenge with tumor cells. Intratumoral GET of two cytokines, one for in situ vaccination and one for an immune boost, proved feasible and effective in eliciting a potent and durable antitumor response; therefore, further studies of this approach are warranted.

## Introduction

Gene electrotransfer (GET) is one of the most efficient non-viral gene therapy approaches that has already reached clinical evaluation (https://clinicaltrials.gov). Using this approach, genetic material encoded on plasmid vectors can be transferred across the cell membrane by applying electric pulses to the system (i.e., electroporation) [1]. In addition to a good safety profile, one of the advantages of GET is its ability to deliver genes directly into various target tissues, including tumors.

Since it allows for local delivery, GET has proven itself as an especially effective method to deliver different cytokines that are toxic if delivered systemically as recombinant proteins. This is especially useful for interleukin 12 (IL-12), which demonstrated efficacy when delivered by GET in several preclinical studies [2]. Furthermore, intratumoral GET of IL-12 has been proven efficient in clinical trials for the treatment of skin melanoma metastases [3]. In addition to its use as a single agent, IL-12 GET can also be employed as vaccination adjuvant [4]. In our previous study [5], we designed a fibroblast-specific, antibiotic resistance gene-free IL-12 plasmid that was specifically optimized to serve as an adjuvant for different vaccination protocols. In the vaccination setting, IL-12 can be used either to boost the immune response to standard vaccines [6, 7] or in combination with local ablative therapies, which can basically act as in situ vaccines due to the release of tumor antigens from the dying cells together with “danger signals” [8–10]. Many local ablative therapies have already been proposed for in situ vaccination, from radiotherapy [11] to electrochemotherapy [9]. In this study, we wanted to test if local ablative therapy via GET of a gene encoding a cytotoxic product could also be used for in situ vaccination in combination with IL-12 boost.

One gene encoding a cytokine with cytotoxic action is *TNFα* [12]. Like IL-12, TNFα is a proinflammatory and immunostimulatory cytokine, but in contrast to IL-12, its antitumor activity is based primarily on its direct cytotoxicity to tumor cells and vascular disruption effects [13]. Due to the significant systemic toxicity, recombinant TNFα is currently used in the clinical practice in the setting of isolated limb perfusion only [14]. Tumor-targeted delivery of TNFα has been attempted by gene therapy approaches [15, 16]. Among them, the most promising was TNFerade (GenVec Inc.), a TNFα-expressing adenovirus vector, which has progressed to a phase III clinical trial for pancreatic cancer patients; however, the efficacy was too low for it to become a viable option [15]. An alternative option to localize the potent effects of TNFα would be to use GET, which, to our knowledge, has never been attempted before. GET of a TNFα plasmid should provide localized effectiveness without systemic toxicity and could, therefore, be used for in situ vaccination, similar to other ablative therapies. Since GET has the capacity to deliver multiple plasmids at once, in the current study our aim was to test the feasibility and effectiveness of the concomitant intratumoral GET of cytokines: TNFα to induce in situ vaccination, and IL-12 to boost the primed local immune response into a systemic one.

## Materials and methods

### Plasmids

Two therapeutic plasmids were used in the study; pORF9 mTNF α, and pCol-mIL-12-ORT. In addition, an empty plasmid, pControl, was used as a control plasmid. pORF9 mTNF α is a commercially available plasmid (Invivogen, Toulouse, France) encoding the mouse *Tnfα* gene under the transcriptional control of the *EF-1a*/*HTLV* hybrid promoter. pCol-mIL-12-ORT encodes the mouse *Il-12* fusion gene under the control of a fibroblast-specific promoter. The plasmid lacks an antibiotic resistance gene. It was prepared in our laboratory using molecular cloning techniques and antibiotic-free ORT technology. Its construction and in vitro evaluation were described in greater detail in our previous study [5]. pControl is also an in-house plasmid [17, 18] and contains only the bacterial backbone.

Plasmids were isolated from bacterial culture using an EndoFree Plasmid Mega kit (Qiagen, Hilden, Germany) and eluted in endotoxin-free water (Qiagen) to a concentration of 1 or 2 mg/mL. The purity and yields were determined spectrophotometrically (Epoch Microplate Spectrophotometer, Take3™ Micro-volume Plate, BioTek, Bad Friedrichshall, Germany). Prior to experiments, the concentration and identity of plasmids were confirmed by restriction analysis on an electrophoretic gel.

### Mouse and tumor models

Tumors were induced in female C57BL/6 mice (Envigo, Udine, Italy) by a subcutaneous injection of 1 × 10^6^ viable B16-F10 cells (American Type Culture Collection, Manassas, VA, USA) in 0.1 mL of saline solution into the right flanks of the mice. When tumors reached 40 mm^3^ mice were randomly divided into different treatment groups and subjected to a specific experimental protocol. Mice were humanely sacrificed when tumor volume reached approximately 300 mm^3^. Mice with complete responses were subjected to secondary challenge with an injection of 1 × 10^6^ viable B16-F10 cells in 0.1 mL of saline solution in the opposite (i.e., left) flank 90 days after tumor remission.

### Gene electrotransfer

Gene electrotransfer (GET) was performed on isoflurane anesthetized mice by intratumoral injection of plasmid DNA followed by an application of electric pulses. Four different plasmid mixtures were reconstituted in 50 µL of endotoxin-free water as follows: a mixture containing 50 µg (19 pmol) of pCol-mIL-12-ORT plasmid DNA; a mixture containing 50 µg (20 pmol) of pORF9 mTNF α; a mixture containing both 50 µg of pCol-mIL-12-ORT and 50 µg of pORF9 mTNF α (39 pmol in total); and a mixture containing 50 µg (22 pmol) of pControl plasmid DNA. To perform GET, 50 µL of plasmid DNA mixture was injected intratumorally, and the tumors were placed between two parallel stainless steel electrodes with a 6 mm gap. Electric pulses (i.e., 8 square-wave pulses with an amplitude of 240 V (600 V/cm, 5 ms, 1 Hz), a duration of 5 ms, and a frequency 1 Hz) were applied by an Electro Cell B10 electric pulse generator (Leroy Biotech, St-Orens-de-Gameville, France) in two perpendicular directions. Endotoxin-free water (50 µL) was used instead of plasmid DNA in the mock GET and control group.

### RT-PCR determination of IL-12 and TNFα expression

To determine IL-12 and TNFα expression, tumors were collected 2 days after GET from 4 to 6 mice per experimental group. RNA was extracted from frozen tumor samples using TRIzol Reagent (Invitrogen, Thermo Fisher Scientific, Waltham, MA, USA) and RNeasy columns (Qiagen, Hilden, Germany), and transcribed into cDNA using the SuperScript VILO cDNA Synthesis Kit (Thermo Fisher Scientific). The 10× diluted mixtures of transcribed cDNA were used as a template for RT-PCR using SYBR® Green Real-Time PCR Master Mix (Thermo Fisher Scientific), which contained a primer mix, SYBR Green (Thermo Fisher Scientific) and DEPC H_2_O (Ambion, Thermo Fisher Scientific). Primers were designed using IDT Primer Quest software (Integrated DNA Technologies, Coralville, IA, USA). Primer pairs for *TNFα* were as follows: forward, TTGTCTACTCCCAGGTTCTCT; reverse, GAGGTTGACTTTCTCCTGGTATG. Primer pairs for *IL-12* were as follows: forward, AGCACGGCAGCAGAATAAA; reverse, CTCCACCTGTGAGTTCTTCAAA. Hypoxanthine–guanine phosphoribosyl transferase (HPRT) was used as a reference gene with the following primer pair: forward, GATTAGCGATGATGAACCAGGTT; reverse, CCTCCCATCTCCTTCATGACA. RT-PCR was performed on a 7300 System (Applied Biosystems). The thermal cycle protocol consisted of activation of uracil-DNA glycosylase (2 min at 50 °C), hot start activation of AmpliTaq Gold Enzyme (10 min at 95 °C), and 45 cycles of denaturation (15 s at 95 °C), annealing and extension (1 min at 60 °C). The 7300 System SDS software (Applied Biosystems) was used for RT-PCR product analysis. Relative quantification of the RT-PCR data was performed using the ${2^{ - \Delta \Delta {C_{\text{t}}}}}$ method.

### Tumor growth delay and survival

The therapeutic effectiveness of GET was determined using a tumor growth delay assay with 4–6 mice per group. Experiments were repeated twice. Tumors were measured by digital Vernier caliper in three perpendicular directions (a, b, c) every 2–3 days. Tumor volume was calculated by the following formula: *V* = *a* × *b* × *c* × *π*/6. From the tumor volumes, arithmetic means for each group were calculated, and tumor growth curves were drawn with error bars representing the standard error of the mean. A Kaplan–Meier survival plot was constructed with a tumor volume of 300 mm^3^ representing the endpoint event. Animals with tumors in regression were examined weekly for tumor presence for 90 days. In addition, the weight of the mice was monitored as a general index of systemic toxicity. The animals were considered cured if they were tumor free at day 90. Cured mice were challenged with a secondary injection of the tumor cells as described above.

### Sample collection and preparation

A set of three mice from each experimental group was reserved for immunological evaluation. Blood, tumors and sentinel lymph nodes were collected 4 days after the last GET. Tumors were fixed in zinc fixative (BD Biosciences, Franklin Lakes, NJ, USA) and embedded in paraffin. The peripheral blood was collected in EDTA-treated tubes (BD Microtainer® MAP) and peripheral blood mononuclear cells (PBMC) were isolated using Ficoll-Paque gradient fractionation. Blood was diluted with PBS at a 1:1 ratio, spun down and gently layered over the Ficoll-Paque™ PLUS (GE Healthcare Life Sciences) in 15 mL conical tubes. The tubes were centrifuged for 30 min at 400×*g* at room temperature. Lymph nodes were passed through a sieve to obtain single-cell suspensions in PBS. Erythrocytes were removed from both types of samples (PBMC and lymph node cell suspension) using a lysing buffer (ACK, Lonza, Basel, Switzerland). Cells were resuspended in cell freezing media (BI Biological Industries, Kibbutz Beit Haemek, Israel) and frozen at − 80 °C for the granzyme B ELISpot assay.

### Histology

The paraffin-embedded tumor samples were cut into 2 µm thick sections from the middle of each block. Four sections were stained using hematoxylin and eosin (H&E) according to standard histochemical procedures. Four sections were used for immunohistochemical determination of granzyme B-positive cells by staining with a granzyme B antibody (ab4059, Abcam, Cambridge, UK) at a 1:1250, and IL-12 and TNFα positive cells by staining with anti-IL12A antibody (ab203031, Abcam) at the dilution 1:1000, and anti-TNFα antibody (ab6671, Abcam) at the dilution 1:1300 overnight at 4 °C. Peroxidase-conjugated streptavidin–biotin kits (ab64261, rabbit-specific HRP/DAB detection IHC kit (ABC), EXPOSE Rabbit-specific HRP-AEC detection IHC kit, Abcam) were used as the colorogenic reagents. Representative images of histological slides were captured by a DP72 CCD camera (Olympus, Tokyo, Japan) connected to a BX-51 microscope (Olympus).

### Granzyme B ELISpot

A Mouse Granzyme B ELISpot kit (R&D Systems, Minneapolis, MN, USA) was used to detect granzyme B-positive cells in blood and lymph node samples. Frozen PBMC and lymph node cells were thawed in a 37 °C water bath, washed and transferred to 50 mL conical tubes containing 5 mL of warm Roswell Park Memorial Institute (RPMI) cell media (Gibco, Thermo Fisher Scientific) containing HEPES (4-(2-hydroxyethyl)-1-piperazine ethanesulfonic acid, Gibco) and 10% fetal bovine serum (Gibco) and were cultured overnight in a humidified 37 °C CO_2_ incubator with loosened caps. The following day cells were washed, counted and resuspended in culture media. In each well of the ELISpot 96-well plate (4 wells per sample), 5 × 10^4^ of cells were plated. To stimulate the production of granzyme B, 500 B16-F10 tumor cells were added to half of the wells (2 wells per sample). ELISpot plates were incubated in a humidified 37 °C CO_2_ incubator for 2 days. The plates were then washed to remove the cells, labelled with the secondary antibody and developed according to the manufacturer’s instructions. Dried plates were imaged and spots were counted. For each sample, the number of granzyme B-positive cells was calculated as the number of spots in the stimulated wells minus the number of spots in the unstimulated wells.

### Statistical analysis

SigmaPlot Software (Systat Software, San Jose, CA, USA) was used for statistical analyses, as well as for graphical representations. All data were tested for normality of distribution with the Shapiro–Wilk test, which failed (*P* < 0.050). Differences between the experimental groups were evaluated by the Kruskal–Wallis one-way analysis of variance on ranks followed by multiple comparisons vs. a control group by Dunn’s method to isolate the groups that were different from the others. A *P* value of less than 0.05 was considered statistically significant.

## Results

**Fig. 1:**
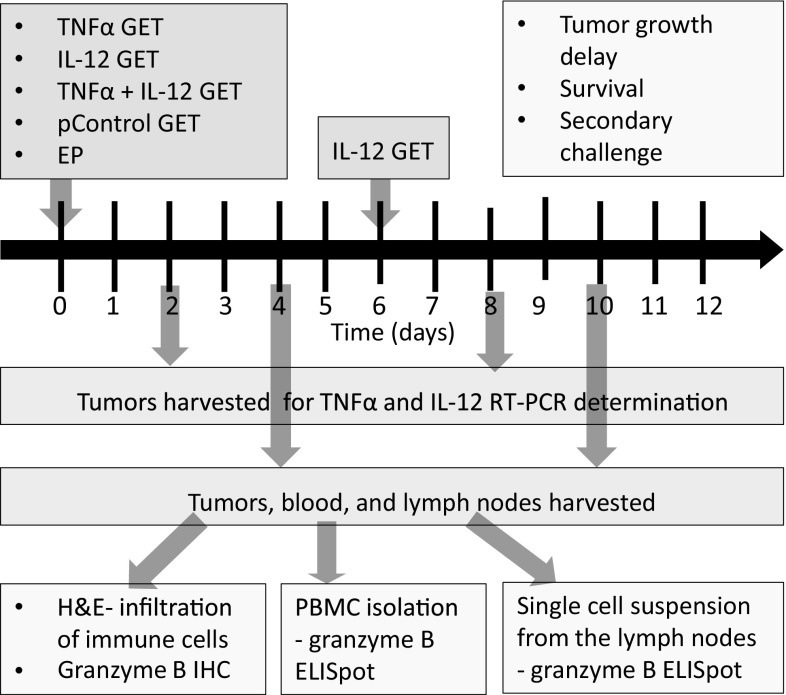
Experimental design. The tested therapeutic groups were as follows: TNF, gene electrotransfer (GET) of the TNFα plasmid; IL-12 GET, GET of the IL-12 plasmid repeated twice with an interval of 6 days; and TNF + IL-12, concomitant GET of the TNFα and IL-12 plasmids, followed 6 days later by GET of the IL-12 plasmid. Additional control groups were as follows: *CTRL* complete control, *EP* electroporation only, *pControl* GET of a control plasmid. On days 2 and 8 tumors were harvested for determination of TNFα and IL-12 expression by RT-PCR. Tumor growth was monitored until the tumor reached a volume of 300 mm^3^, which was also used as the endpoint event for plotting the Kaplan–Meier survival curve. Mice that were tumor free for 90 days were subjected to secondary challenge with an injection of tumor cells. Tumors, blood and lymph nodes were collected on days 4 and 10. Tumors were used for histological determination of immune cell infiltration (H&E, hematoxylin and eosin staining) and immunohistochemical (IHC) determination of granzyme B-positive cells. Granzyme B ELISpot was performed on the PBMC and single-cell suspensions isolated from the lymph nodes

This study examined the concomitant intratumoral GET of TNFα and IL-12 plasmids. The tested treatments were: TNFα GET monotherapy (TNF), interleukin 12 monotherapy (IL-12) that consisted of two IL-12 GET treatments performed twice, with an interval of 6 days, and a combination in which both cytokines were first delivered concomitantly in one GET session, followed 6 days later by IL-12 GET (TNFα + IL-12) (Fig. 1). The feasibility and effectiveness of the treatments were determined by measuring the expression of delivered plasmids, and by assessing tumor growth delay and survival. The activation of immune response was measured by monitoring tumor take after secondary challenge and by analyzing tumor infiltration by immune cells and the expansion of effector immune cells in the sentinel lymph nodes and blood.

### Expression of plasmids in the tumors

**Fig. 2:**
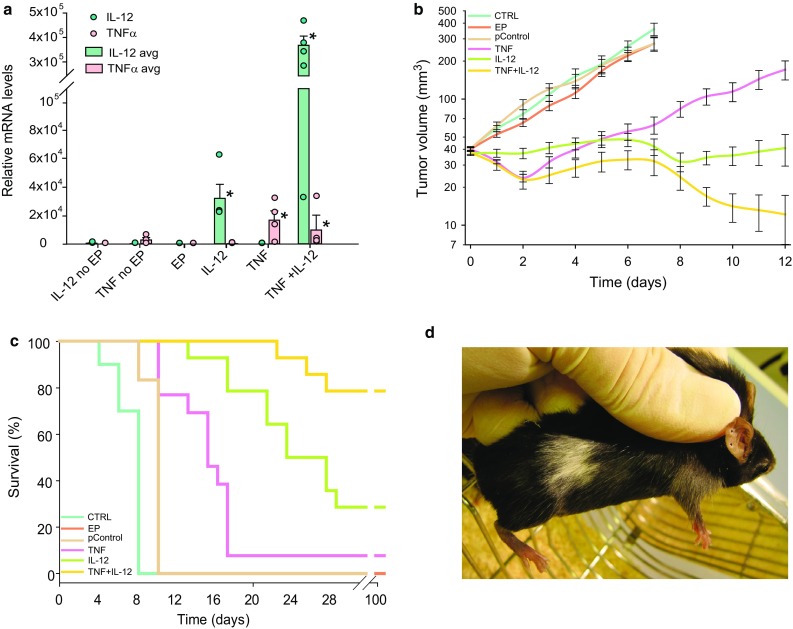
Expression and therapeutic effectiveness after TNFα and IL-12 GET in B16-F10 murine melanoma tumors. **a** Expression of IL-12 and TNFα as determined by RT-PCR 2 days after GET. Circles represent expression in the individual tumors; Bars represent the mean level of expression with the standard error of the mean of 5 mice per experimental group. **b** Growth of the treated tumors. Data were pooled from 2 independent experiments with 5–6 animals in each experimental group and are presented as the mean with the standard error of the mean. A tumor volume of 300 mm^3^ was pre-set as the humane endpoint of the experiment. **c** Survival of the treated animals. A Kaplan–Meier survival plot was constructed with a tumor volume of 300 mm^3^ counted as the endpoint event. **d** Vitiligo, local discoloration of the fur at the treatment area, observed in all the cured mice. *EP* application of electroporation only, *IL-12 no EP* injection of IL-12 plasmid without EP, *TNF no EP* injection of TNFα plasmid without EP, *TNF* TNFα GET, *IL-12* IL-12 GET (repeated twice), *TNF + IL-12* TNFα and IL-12 GET, *pControl* GET of a control plasmid (pControl)

**Fig. 3:**
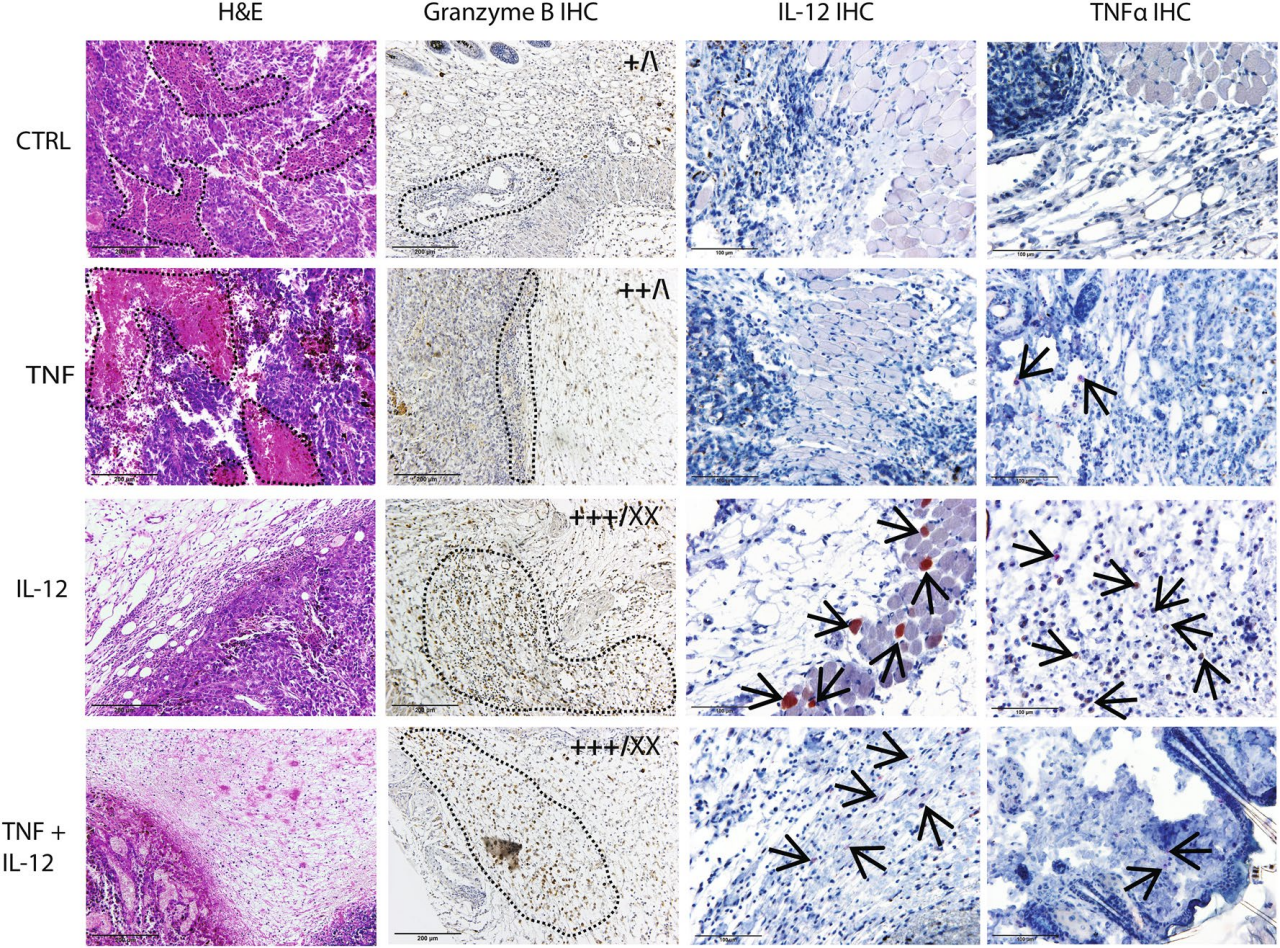
Representative images of histological tumor sections in the control group (CTRL), after TNFα (TNF) and IL-12 (IL-12) GET, and after GET of both plasmids (TNF + IL-12). Sections were stained with hematoxylin and eosin (H&E, first column) or for granzyme B (second column), IL-12 (third column), and TNFα (forth column) positive cells. The infiltration of immune cells and the abundance of granzyme B-positive cells were scored using a semi-quantitative method. Scoring results are shown on the upper right corner of the representative images as follows: left, infiltration; right, granzyme B-positive cells. **– – –**, necrosis in column 1; lymphocyte infiltration in column 2; **→**, positive muscles, fibroblasts and macrophages in columns 3 and 4; +, low infiltration; ++, high infiltration; +++, very high infiltration; \, no granzyme B-positive cells; X, low frequency of positive cells; XX, high frequency of positive cells. Images were taken by a DP72 CCD camera (Olympus, Tokyo, Japan) connected to a BX-51 microscope (Olympus)

IL-12 GET was performed twice, with an interval of 6 days. This protocol was chosen based on the results of a pilot study testing the therapeutic effectiveness of the new IL-12 plasmid, which showed that tumors treated with IL-12 monotherapy started to grow back after 6 days (data not shown). After IL-12 GET, IL-12 mRNA levels were significantly increased, by a factor of 23 × 10^3^ after repeated IL-12 GET. After TNFα GET, TNFα mRNA levels also were significantly increased, by a factor of 17 × 10^3^. After concomitant GET of IL-12 and TNFα, mRNA levels of both IL-12 and TNFα were significantly increased, especially the levels of IL-12, which increased by a factor of 364 × 10^3^ (Fig. 2a). In addition, the expression of both cytokines was assessed on the histological section taken 4 days after GET, which showed positive staining for IL-12 of certain muscle fibers and fibroblasts, while IL-12 and TNFα positive macrophages could be found in all groups but were more abundant in the IL-12 monotherapy group (Fig. 3).

### Therapeutic effectiveness

IL-12 GET resulted in a tumor growth delay of 16 days, and 28.6% complete responses. TNFα GET was less effective, having only a moderate effect on tumor growth (tumor growth delay of 6 days) and a low cure rate. However, combined treatment with IL-12 and TNFα resulted in a pronounced tumor growth delay of 27 days (calculated for the 3 mice that did not regress) (Fig. 2b). Furthermore, the combined treatment increased the survival of mice from 28% in IL-12 GET monotherapy to 79%. Rejection after secondary challenge was demonstrated in 100% of the mice in the combined treatment group, and 75% in IL-12 monotherapy group (Fig. 2c). All the cured mice developed vitiligo localized to the treatment area (Fig. 2d).

### Immune response in tumors

H&E staining showed extensive infiltration of lymphocytes in the tumor site in the IL-12 monotherapy and the combined treatment groups (Fig. 3); in fact, in these groups, tumors were mostly absent at the time of sampling and were replaced with lymphocyte infiltrate. Tumor-infiltrating lymphocytes were further characterized by immunohistochemical staining for granzyme B, which marks activated effector lymphocytes. After combined treatment, most tumor-infiltrating lymphocytes were granzyme B positive (Fig. 3), which was also the case in the two tumors that regressed in the IL-12 monotherapy group (Fig. 3). H&E staining also showed extensive necrosis in the control and the TNFα GET groups. Tumors after TNFα GET were smaller and necrosis was more evenly distributed throughout the tumor compared to in the control tumors, where the necrosis was mainly centrally located.

### Immune response in lymph nodes and blood

**Fig. 4:**
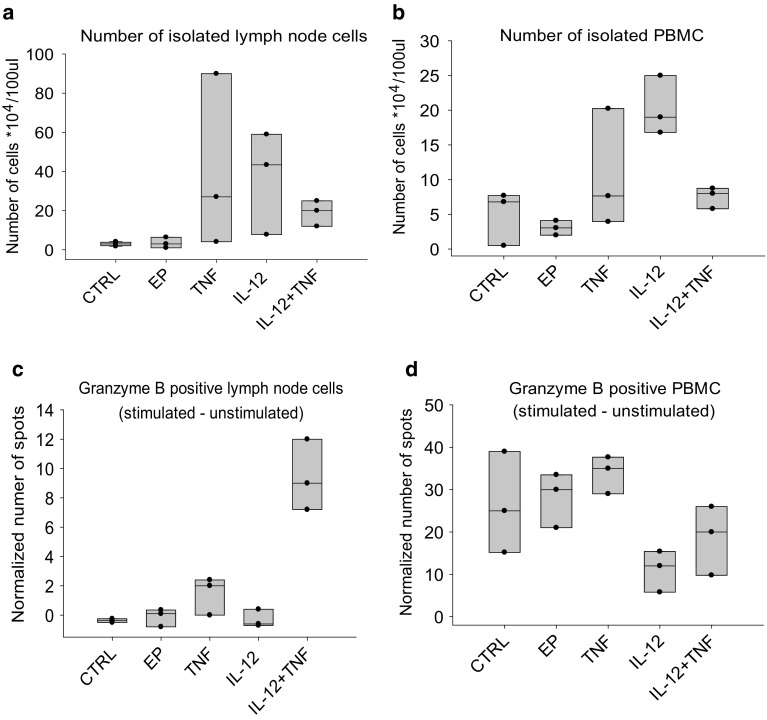
Results of the granzyme B ELISpot performed on single-cell suspensions from the lymph nodes and PBMC 4 days after treatment. **a** Number of cells isolated from the lymph nodes. **b** Number of PBMC isolated from blood. **c** Number of granzyme B-positive cells in the lymph nodes after exposure to tumor cells, normalized to cells that were not stimulated with tumor cells. **d** Number of granzyme B-positive PBMC after exposure to tumor cells, normalized to cells that were not stimulated with tumor cells. Circles represent values for individual samples; boxes show the variance with the median values for three samples per experimental group. *CTRL* control group, *EP* application of electroporation only, *TNF* TNFα GET, *IL-12* IL-12 GET, *TNF + IL-12* TNFα and IL-12 GET

Expansion of the effector immune cells in sentinel lymph nodes and blood was assessed by granzyme B ELISpot, performed on single-cell suspensions isolated from lymph nodes and peripheral blood mononuclear cells (PBMC) 4 days after the completion of therapy. Counting of isolated cells demonstrated that, on average, more cells were present after monotherapies in both lymph nodes (40 × 10^4^/100 mL in TNFα and 36 × 10^4^/100 mL in IL-12 group vs. 3 × 10^4^/100 mL in control) and blood (11 × 10^4^/100 mL in TNFα and 20 × 10^4^/100 mL in IL-12 group vs. 5 × 10^4^/100 mL in control), compared to the combined treatment group, where the cell yields were low in both lymph nodes (19 × 10^4^/100 mL) and in blood (7 × 10^4^/100 mL) (Fig. 4). However, the number of spot-forming cells among the isolated cells was higher after combined treatment, but only in the lymph nodes (9 vs. 0 in the control group) and not in the blood (19 vs. 26 in the control group). The number of granzyme B-positive cells was lowest after IL-12 monotherapy in both lymph nodes (0) and blood (11).

## Discussion

**Fig. 5:**
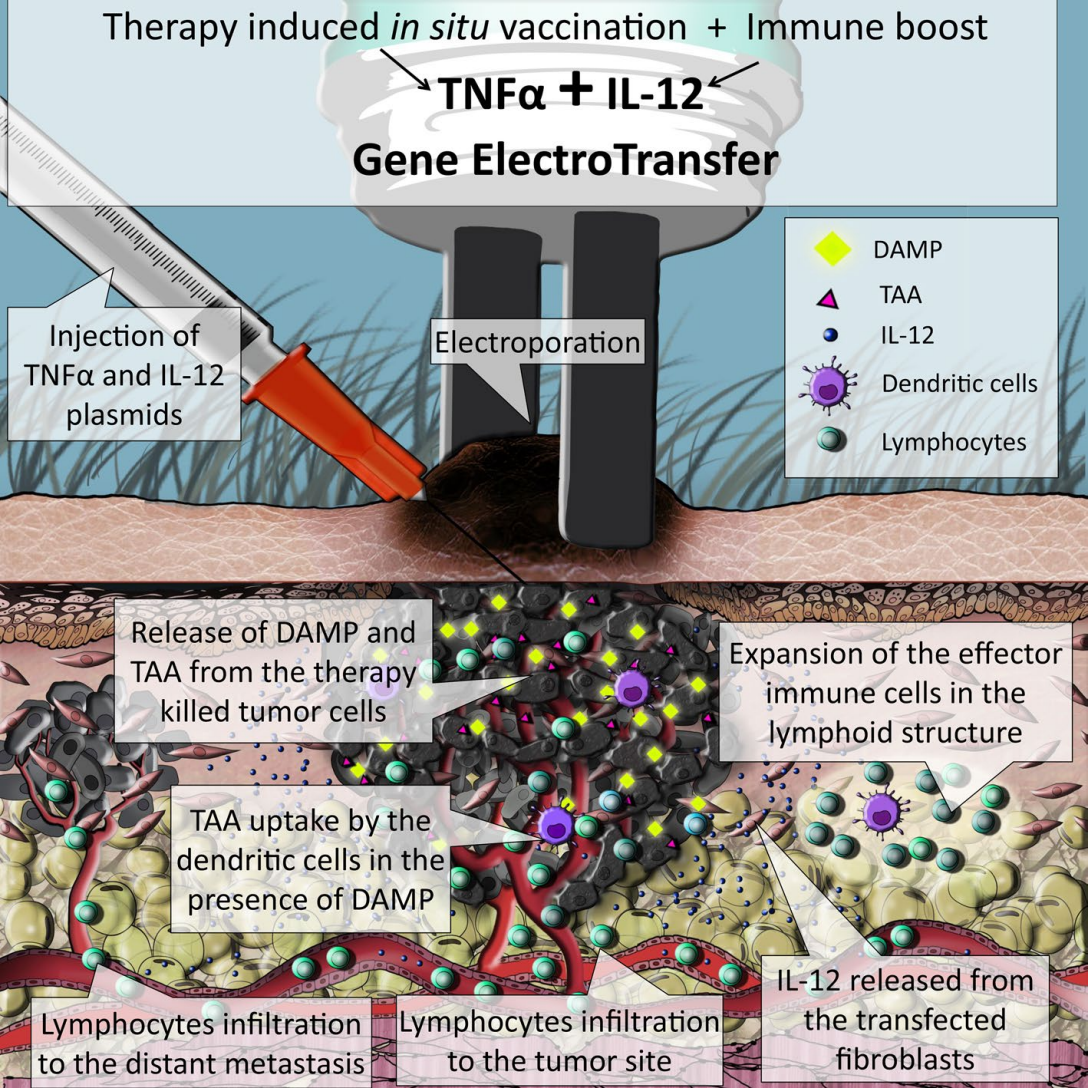
Simulation of the events taking place inside a melanoma tumor treated with concomitant GET of TNFα and IL-12: the TNFα expressed from the transfected cells causes immunogenic form of cell death, accompanied by the release of tumor associated antigens (TAA) together with danger signals (DAMP). Released TAA are then captured by dendritic cells (DC) that are attracted to the tumor site, also by the IL-12 released from the transfected cells. DC migrate to the lymph structures where they initiate expansion of effector immune cells specific for the captured TAA. Activated tumor specific lymphocytes are released to the blood stream and can infiltrate primary tumor and metastases, where they exert their antitumor effects

In this study, we tested the feasibility and effectiveness of in situ vaccination with TNFα and IL-12 by concomitant GET in murine melanoma tumors (Fig. 5). After GET, both TNFα and IL-12 mRNA levels significantly increased, resulting in a pronounced tumor growth delay of 27 days and a prolonged survival rate. Complete responses were seen in 79% of mice and there was a 100% rejection rate after the secondary challenge, as the measure of immune memory. Activation of the antitumor immune response was confirmed by extensive infiltration of immune cells in the tumor site and expansion of the effector immune cells in the sentinel lymph nodes. Furthermore, the effects of in situ vaccination were indicated by the occurrence of fur depigmentation localized to the treatment area, known as vitiligo.

First, we assessed if concomitant delivery of the selected plasmids by GET was feasible by analyzing the expression of the transfected plasmid. Since plasmids have no size limitation, unlike viral vectors, GET has the capacity to deliver multiple genes, either in one large polycistronic plasmid or in multiple normally sized plasmids; however, since larger plasmids are harder to transfect, delivery of multiple plasmids is more reasonable [19]. GET of multiple genes has been previously used for CRISPR/Cas9 technology [20] and for the preparation of induced pluripotent stem cells [21]. Moreover, it has also been used for the combined delivery of DNA vaccines and immune adjuvants [22] and for the delivery of multiple therapeutic genes in vivo [23]. The latter was also confirmed in this study, since the RT-PCR results showed that after GET, both TNFα and IL-12 mRNA levels increased significantly.

The TNFα and IL-12 expression analyses showed some interesting results. After repeated IL-12 GET, the expression of IL-12 not only doubled, but increased by a factor of 5 (from 4 to 23), compared to the expression after single IL-12 GET. Furthermore, after the combined therapy with TNFα, the increase in IL-12 expression was even greater, specifically, by a factor of 16 (from 23 to 364). For the interpretation of these results, it is worth mentioning that the RT-PCR primers were not specifically designed to distinguish plasmid expressed IL-12 from endogenously expressed IL-12. Therefore, we believe that the disproportionately high expression of IL-12 was the result of a positive feedback loop through IFNγ, which led to the production of endogenous IL-12 [24]. This was confirmed on the histological sections stained for IL-12, which showed positive staining of a few muscle fibers and fibroblast and numerous positive macrophages dispersed all around the tumor, or what was left of it. Based on the cell type that stained positively, we could actually distinguish between the endogenously produced IL-12 and the plasmid expressed IL-12, since we know that GET typically results in the transfection of the connective tissue around the tumor and particularly the muscles [40], while the endogenous IL-12 is secreted form the immune cells, like macrophages.

Altogether, the expression results proved that concomitant GET of IL-12 and TNFα is feasible. While IL-12 GET was already tested in numerous studies, this is the first study using GET of TNFα. In general, GET-mediated delivery of cytotoxic genes, so-called “gene chemotherapy” or “suicidal gene therapy”, has not been extensively studied, with the exception of several preclinical studies using a strategy with the herpes simplex virus thymidine kinase (HSV-TK) and prodrug ganciclovir (GCV) [25–27], as well as one study regarding the delivery of tumor necrosis factor-related apoptosis-inducing ligand (TRAIL) [28]. These studies showed that GET-based suicidal gene therapy could provide a potentially effective gene therapy for cancer.

In the present study, TNFα GET was intended as a local ablative therapy that could work as in situ vaccination. TNFα was chosen based on its ability to cause immunologically relevant cell death. Although TNFα was first identified as an agent capable of causing tumor necrosis and was later considered an apoptotic inducer, new evidence suggests that TNFα induces a special form of cell death termed necroptosis that is programmed, but immunogenic [29], and thus suitable for in situ vaccination [30, 31]. However, in our study, TNFα monotherapy was not very successful and led to a tumor growth delay of only 6 days, and 1 complete response that was not permanent, because the secondary challenge resulted in tumor growth. These results confirmed the necessity of combining local ablative techniques with an immune boost. Namely, although local ablative therapies are capable of priming the immune response against released TAA, vaccination-like effects are rare [10, 11, 32]; for instance, the abscopal effect is well documented only after radiotherapy [33], indicating that the primed immune response needs to be boosted.

Indeed, when we combined the TNFα with IL-12 treatment, the therapeutic effectiveness increased significantly, as demonstrated by a pronounced tumor growth delay of 27 days, compared to only 6 days after TNFα monotherapy and 16 days after IL-12 monotherapy. The observed differences in therapeutic effectiveness between monotherapies and combined therapy were reflected in the tumor histology. Tumors after TNFα GET were larger and contained low levels of infiltrating lymphocytes, while after IL-12 GET monotherapy or combined treatment, H&E staining showed extensive infiltration of lymphocytes in the tumor site. Furthermore, after combined treatment, most tumor-infiltrating lymphocytes stained positively for granzyme B. These results are in accordance with a number of reports that demonstrated that tumor infiltration with effector immune cells is one of the most significant positive predictive markers for the success of various anticancer therapies [34, 35].

The presence of effector immune cells was also assessed by granzyme B ELISpot in the blood and lymph node samples. On average, more cells were isolated after both monotherapies than after the combined treatment, where the cell yields were low, probably because the mice in this group were mostly tumor free at the time of analysis, meaning that the acute phase of the immune response was already over. The number of granzyme B-positive cells, however, was higher after the combined treatment, but only in the lymph nodes and not in the blood, confirming the clonal expansion of effector cells inside the lymph nodes and demonstrating that blood is not a good surrogate marker for the effectiveness of immunotherapy, as was already confirmed in the previous studies [36].

Interestingly, the amount of granzyme B-positive cells in the lymph nodes and blood were the lowest after IL-12 GET monotherapy. This could be due to the general activation of the immune system in this group, and not a specific response, given that immune targets (i.e., TAA) were missing, since IL-12 is not directly cytotoxic to tumor cells [37]. In spite of that, GET-mediated monotherapy with IL-12 can be very successful resulting in up to 90% of complete responses in the preclinical melanoma tumor models [38, 39]. The proposed explanation for this observed success is that the antitumor effectiveness of IL-12 GET does not just result from the expressed therapeutic transgene [40, 41], but also from a type 1 interferon immune response, triggered by the introduction of foreign DNA. The plasmid used in this study was specifically designed to minimize these transgene non-specific effects through the removal of a large portion of the bacterial DNA backbone, and through the use of a weaker promoter endogenous to eukaryotic cells [5]. These modifications could explain the lower effectiveness of IL-12 GET monotherapy observed in this study, compared to in similar, previously published studies [38, 39].

In all the cured mice, the effects of in situ vaccination were also indicated by the occurrence of depigmentation of the fur, or vitiligo, localized to the treatment area. The development of vitiligo-like skin depigmentation or melanoma-associated leukoderma has been associated with favorable clinical outcome in patients with metastatic melanoma, especially after immunotherapy, and, recently, also after radiotherapy [18, 42]. This depigmentation is the result of a specific immune response against melanoma antigens that are also present in melanocytes, and it is therefore a sign of the induction of an anti-melanoma immune response. This means that the observed depigmentation in our study can be considered as proof that in situ vaccination can be accomplished by GET of TNFα and IL-12 plasmids.

## Conclusions

Currently, the combination of immune adjuvants and local ablative techniques, which act as in situ vaccines due to their induction of TAA release, is gaining much attention. In this study, we tested a simplified version of this approach by utilizing the concomitant intratumoral GET of two plasmids. For in situ vaccination, a plasmid encoding TNFα was used, while a plasmid encoding IL-12 was used for an immune boost. The results confirmed the feasibility and effectiveness of the proposed approach in eliciting a potent and durable antitumor response. The ability of this approach to induce in situ vaccination was indicated by the expansion of effector immune cells in the lymph nodes, and vitiligo-like depigmentation of the treated area. However, further studies are needed to directly prove the systemic effectiveness (i.e., abscopal effect) of the approach and to push the local effectiveness from 80 to 100%. In addition, the approach needs to be tested in other tumor models, especially since one of the advantages of in situ vaccination is its ability to harnesses the patient’s own immune system and tumors own TAA and consequently has the potential to be effective in different cancer types.

## Abbreviations

DAMP: Damage-associated molecular patterns
GET: Gene electrotransfer
IL-12: Interleukin 12
TNFα: Tumor necrosis factor alpha
